# Editorial: Remedying the injured brain in cognitive impairment: potential neuroimmune communication signaling and therapeutic opportunities

**DOI:** 10.3389/fimmu.2025.1705628

**Published:** 2025-09-30

**Authors:** Zhengsen Jin, Zhongdi Cai, Asma Perveen, George E. Barreto, Linlin Yin, Rong Wang, Rui Liu

**Affiliations:** ^1^ Institute of Medicinal Biotechnology, Peking Union Medical College and Chinese Academy of Medical Sciences, Beijing, China; ^2^ Department of Biochemistry, Faculty of Life Sciences, Aligarh Muslim University, Aligarh, India; ^3^ Glocal School of Life Sciences, Glocal University, Saharanpur, Uttar Pradesh, India; ^4^ Department of Biological Sciences, University of Limerick, Limerick, Ireland; ^5^ Department of Neuroinfection and Neuroimmunology, Beijing Tiantan Hospital, Capital Medical University, Beijing, China; ^6^ Central Laboratory, Xuanwu Hospital, Capital Medical University, Beijing, China

**Keywords:** Alzheimer’s disease, central nervous system disorders, microglia, multiple sclerosis, neuroimmune signaling, neuroinflammation, therapeutic strategies

Nearly 16% of the global population suffers from neurological disorders linked to acute or chronic inflammation, including neurodegenerative and cerebral neuroimmune diseases (1, 2). Neuroimmune dysregulation is widely acknowledged as a pivotal mechanism in the pathogenesis of these disorders, involving intricate immune responses and cellular interactions within the central nervous system (CNS) (3). Recent advances at the intersection of neuroscience and immunology have greatly enhanced our understanding of neuroimmune signaling pathways (4). Studies have shown that resident immune cells in the brain, such as microglia and astrocytes, together with peripheral immune cells, including T cells, B cells, and monocytes, play critical roles in neuroinflammation, vascular injury, and cognitive decline (5–7). For instance, the functional states of microglia, such as the pro-inflammatory M1-type and neuroprotective M2-type polarization, as well as their transformation into disease-associated microglia, coupled with aberrant activation of the complement system, are significantly implicated in the progression of neurodegenerative diseases (8). Furthermore, the infiltration of peripheral immune cells and their communication with the CNS remain major themes in neuroimmune research (9, 10). This Research Topic brings together cutting-edge advances in neuroimmune signaling, novel microglial polarization states, peripheral immune system-to-CNS immune crosstalk, and multi-omics biomarkers associated with cognitive impairment and dementia, with a special focus on innovative therapeutic strategies targeting neuroimmune inflammation. The Research Topic includes six papers: four original research papers, one systematic review, and one opinion piece.

## Advanced insights into neuroimmune communication signaling in neurological disorders

Advances in sequencing technologies, particularly epitranscriptomic and single-cell sequencing (scRNA-seq), have profoundly improved our understanding of brain cell types, states, and regulatory mechanisms (11, 12). Epigenetic dysfunction leading to altered gene expression in the brain is recognized as a key pathophysiological feature of aging-related neurodegeneration (13). N6-methyladenosine (m^6^A) RNA methylation and demethylation, the most prevalent internal RNA modifications in eukaryotes, regulate multiple aspects of RNA metabolism and associated signaling pathways (14). Given the strong genetic component of Alzheimer’s disease (AD) and related dementias, Liao et al. demonstrated that chronic intermittent ethanol exposure reduced Th2 immune cells in the brains of AD mice. Using Methylated RNA Immunoprecipitation sequencing (MeRIP-seq) and RNA sequencing (RNA-seq), they further revealed that increased expression of RNA binding motif protein 15B (Rbm15b) and decreased expression of heterogeneous nuclear ribonucleoprotein A2/B1 (Hnrnpa2b1) contributed to synaptic dysfunction and neuroinflammation underlying memory impairment. This study provides an epigenetic link between chronic alcohol consumption and cognitive deficits in AD mice. Beyond inflammation triggered by chronic external factors, intrinsic immune signaling pathways also play crucial roles in AD. Li et al. identified dysregulated expression of multiple NOD-like receptor (NLR) family members in hippocampal RNA-seq data from AD patients. They further confirmed significant upregulation of NLR family pyrin domain-containing 3 (NLRP3) in microglia surrounding amyloid-β plaques in both AD mice and human postmortem tissue. Functional studies of NLRP3 could reveal novel therapeutic avenues for AD. Apart from microglia-centered mechanisms in AD, high-throughput technologies are also being used to identify regulatory targets in other neuroimmune disorders. Yu et al. utilized scRNA-seq to examine transcriptional states and cellular composition of peripheral blood mononuclear cells in multiple sclerosis (MS) patients. Integrated with multiple Mendelian randomization approaches, they identified Fc receptor-like 3 (FCRL3), a B cell-specific receptor, as a potential protective immune node, offering a fresh perspective on immunoregulatory mechanisms in MS.

## Therapeutic opportunities targeting neuroimmune pathways in neurological disorders

This Research Topic also highlights therapeutic strategies aimed at immune-inflammatory mechanisms in neurological diseases. Among the myriad of inflammatory mediators, microRNAs (miRNAs), a subset of small non-coding RNAs, are recognized as key regulators of numerous biological processes, owing to their ability to modulate multiple genes simultaneously (15). miRNAs are highly abundant in the CNS, with each cell type exhibiting a distinct expression profile (16). Elucidating the spatiotemporal regulation of miRNAs and their targets under pathological conditions is essential for developing miRNA-based biomarkers and therapeutic interventions. In this Research Topic, Zhao et al. emphasize the role of a novel miRNA, miR-25802, in AD-related neuroinflammation. They demonstrated that miR-25802 upregulation coincides with early microglial activation in AD, suggesting its diagnostic potential. Mechanistically, miR-25802 directly binds to and suppresses Krüppel-like transcription factor 4 (*KLF4*) mRNA, promoting microglial polarization toward a pro-inflammatory M1 phenotype via nuclear factor κB (NF-κB)-mediated inflammation. The authors also proposed that using AAV9-based delivery of regulators targeting the miR-25802/KLF4/NF-κB axis could offer new treatment strategies for AD. Given the central role of NF-κB in inflammation, Bai et al. conducted a comprehensive bibliometric analysis of 1,468 publications (2008–2023) to delineate the research landscape of NF-κB in cognitive impairment. Their findings identified current and emerging research themes, including neuroinflammation, microglial activation, and NF-κB-mediated signaling networks. The study also noted growing interest in apolipoprotein E, gut microbiota, and ketogenic diet-based interventions, reflecting a shift toward multidisciplinary treatment approaches. As the resident immune cells of the CNS, microglia also participate in neurogenesis, modulation of synaptic plasticity, neuronal network maintenance, and contribute to synapse formation and pruning through the elimination of inactive, weak, or excessive synapses (17). This clearance process depends on microglia triggering receptor expressed on myeloid cells 2 (TREM2), C1q and C3 proteins, and the phagocytic capacity of microglia (18). Zhang et al. revealed that dysregulated phagocytosis in prelimbic microglia affected spine density and glutamatergic signaling, thereby influencing the formation of fear memory. In a fear conditioning model, TREM2 knockout restored synaptic homeostasis, enhanced neuronal function, and improved memory performance, suggesting that targeting TREM2 to modulate microglial activity may represent a promising therapeutic strategy.

Overall, this Research Topic elucidates the critical roles and underlying mechanisms of neuroimmune dysregulation in various CNS disorders, including AD, cognitive impairment, MS, and fear-related psychiatric disorders. With accumulating evidence implicating immune-inflammatory processes in disease progression, we aimed to spotlight advances in neuroimmune signaling, multi-omics biomarkers, and novel therapeutic strategies. The findings underscore the potential of targeting neuroimmune crosstalk, such as through TREM2 modulation, NLRP3 inhibition, or miRNA-based therapies, to alleviate neuroinflammation and cognitive decline. We hope this Research Topic inspires further research into immune-focused diagnostics and treatments, ultimately contributing to effective interventions for neuroinflammatory and neurodegenerative diseases. Finally, we extend our sincere gratitude to the authors, reviewers, and the Editorial Office of Frontiers in Immunology for their outstanding contributions and dedicated support of this Research Topic.

## References

[1] Shi FD Yong VW. Neuroinflammation across neurological diseases. Science. (2025) 388:eadx0043. doi: 10.1126/science.adx0043, PMID: 40536983

[2] Wilson DM 3rd Cookson MR Van Den Bosch L Zetterberg H Holtzman DM Dewachter I. Hallmarks of neurodegenerative diseases. Cell. (2023) 186:693–714. doi: 10.1016/j.cell.2022.12.032, PMID: 36803602

[3] Wheeler MA Quintana FJ. The neuroimmune connectome in health and disease. Nature. (2025) 638:333–42. doi: 10.1038/s41586-024-08474-x, PMID: 39939792 PMC12039074

[4] Akassoglou K Davalos D Mendiola AS Petersen MA Ryu JK Schachtrup C. Pioneering discovery and therapeutics at the brain-vascular-immune interface. Cell. (2024) 187:5871–6. doi: 10.1016/j.cell.2024.09.018, PMID: 39423805 PMC13046387

[5] Sepehrinezhad A Gorji A. Pericyte-glial cell interactions: Insights into brain health and disease. Neural Regener Res. (2026) 21:1253–63. doi: 10.4103/NRR.NRR-D-24-01472, PMID: 40537003 PMC12407509

[6] Li P Huang C He Y Lei X Shen X Xu J. M2 macrophage-laden vascular grafts orchestrate the optimization of the inflammatory microenvironment for abdominal aorta regeneration. Acta Biomater. (2025) S1742-7061:00598–7. doi: 10.1016/j.actbio.2025.08.016, PMID: 40784447

[7] Wang Y Zhang M Zhang T Zhang S Ji F Qin J. PD-L1/PD-1 checkpoint pathway regulates astrocyte morphogenesis and myelination during brain development. Mol Psychiatry. (2025) 30:3895–911. doi: 10.1038/s41380-025-02969-3, PMID: 40164696

[8] Mrdjen D Cannon BJ Amouzgar M Kim Y Liu C Vijayaragavan K. Spatial proteomics of Alzheimer’s disease-specific human microglial states. Nat Immunol. (2025) 26:1397–410. doi: 10.1038/s41590-025-02203-w, PMID: 40696045 PMC13075585

[9] Van Hove H De Feo D Greter M Becher B. Central nervous system macrophages in health and disease. Annu Rev Immunol. (2025) 43:589–613. doi: 10.1146/annurev-immunol-082423-041334, PMID: 40036702

[10] Kaur R Kumar S Singh L. A comprehensive review: neuroinflammation and immune communication between the central nervous system and the periphery. Cytokine. (2025) 192:156974. doi: 10.1016/j.cyto.2025.156974, PMID: 40449035

[11] Moquin-Beaudry G Andriamboavonjy L Audet S Hamilton LK Duquette A Chouinard S. Mapping the peripheral immune landscape of Parkinson’s disease patients with single-cell sequencing. Brain. (2025) 148:2847–60. doi: 10.1093/brain/awaf066, PMID: 40417848 PMC12316021

[12] Jiang J Zhang M Xia W Ding C Li J Hu X. Fto-mediated m6A modification is essential for cerebellar development through regulating epigenetic reprogramming. J BioMed Sci. (2025) 32:81. doi: 10.1186/s12929-025-01176-0, PMID: 40883822 PMC12398073

[13] Hwang JY Aromolaran KA Zukin RS. The emerging field of epigenetics in neurodegeneration and neuroprotection. Nat Rev Neurosci. (2017) 18:347–61. doi: 10.1038/nrn.2017.46, PMID: 28515491 PMC6380351

[14] An Y Duan H. The role of m6A RNA methylation in cancer metabolism. Mol Cancer. (2022) 21:14. doi: 10.1186/s12943-022-01500-4, PMID: 35022030 PMC8753874

[15] Zhao K Liu J Sun T Zeng L Cai Z Li Z. The miR-25802/KLF4/NF-κB signaling axis regulates microglia-mediated neuroinflammation in Alzheimer’s disease. Brain Behav Immun. (2024) 118:31–48. doi: 10.1016/j.bbi.2024.02.016, PMID: 38360375

[16] Zeng L Cai Z Liu J Zhao K Liang F Sun T. miR-32533 reduces cognitive impairment and amyloid-β Overload by targeting CREB5-mediated signaling pathways in alzheimer’s disease. Adv Sci (Weinh). (2025) 12:e2409986. doi: 10.1002/advs.202409986, PMID: 39840513 PMC11905094

[17] Marques SI Sá SI Carmo H Carvalho F Silva JP. Pharmaceutical-mediated neuroimmune modulation in psychiatric/psychological adverse events. Prog Neuropsychopharmacol Biol Psychiatry. (2024) 135:111114. doi: 10.1016/j.pnpbp.2024.111114, PMID: 39111563

[18] Park JC Han JW Lee W Kim J Lee SE Lee D. Microglia Gravitate toward Amyloid Plaques Surrounded by Externalized Phosphatidylserine via TREM2. Adv Sci (Weinh). (2024) 11:e2400064. doi: 10.1002/advs.202400064, PMID: 38981007 PMC11425970

